# Symbiont Reintroduction Alters Tumor Progression and Life‐History Traits in the Tumor‐Bearing Freshwater Cnidarian *Hydra oligactis*

**DOI:** 10.1002/ece3.73458

**Published:** 2026-04-13

**Authors:** Nikita Stepanskyy, Jordan Meliani, Jácint Tökölyi, Aurora M. Nedelcu, Beata Ujvari, Frédéric Thomas, Antoine M. Dujon

**Affiliations:** ^1^ CREEC/CANECEV (CREES), MIVEGEC Unité Mixte de Recherches, IRD 224–CNRS 5290, Université de Montpellier Montpellier France; ^2^ MTA‐DE “Momentum” Ecology, Evolution and Developmental Biology Research Group, Department of Evolutionary Zoology University of Debrecen Debrecen Hungary; ^3^ Department of Biology University of New Brunswick Fredericton New Brunswick Canada; ^4^ Centre for Integrative Ecology, School of Life and Environmental Sciences Deakin University Waurn Ponds Victoria Australia

**Keywords:** biotic interactions, commensalism, host–symbiont dynamics, neoplasm, tumor ecology

## Abstract

Environmental changes can disrupt long‐standing host–symbiont associations and influence tumor dynamics; however, how these two aspects interact remains poorly understood, particularly when previously co‐evolved symbionts are reintroduced into tumor‐prone hosts. We experimentally reintroduced a native commensal ciliate symbiont (*Kerona pediculus*) into two long‐term cultured symbiont‐free lines of the freshwater cnidarian, *Hydra oligactis* , differing in tumor affliction: one harbors a transmissible tumor, and one has historically low spontaneous tumor incidence. Unexpectedly, spontaneous tumors emerged at high frequency in the latter, independently of ciliate acquisition, fundamentally reshaping the experimental framework and enabling comparisons of how symbiont reintroduction affects hosts with either transmissible or *de novo* tumors. While ciliate infection did not alter tumor incidence, it slightly accelerated tumor onset, increased the likelihood of supernumerary tentacle formation, and reduced asexual reproduction (particularly at high symbiont densities) across tumor contexts. Spontaneous tumors appeared later than transmissible tumors, were less often associated with supernumerary tentacles, and induced an earlier reproductive burst. Our findings show that symbiont reintroduction and tumor context shape tumor dynamics and life‐history traits in tumor‐bearing hosts, emphasizing the potential role of symbiotic history and tumor evolutionary context when assessing the outcomes of such pressures in vulnerable host populations.

## Graphical abstract

We reintroduced the native ciliate *Kerona pediculus* into long‐term symbiont‐free *Hydra oligactis* lines differing in tumor affliction. Unexpectedly, spontaneous tumors arose at high frequency independently of ciliate acquisition, fundamentally reshaping the experimental framework and enabling comparisons of how symbiont reintroduction affects hosts with either transmissible or *de novo* tumors. Our findings show that symbiont reintroduction and tumor context shape tumor dynamics and life‐history traits in tumor‐bearing hosts, emphasizing the potential role of symbiotic history and tumor evolutionary context when assessing the outcomes of such pressures in vulnerable host populations.

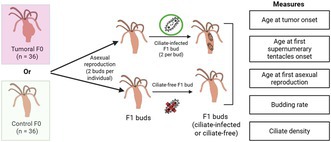

## 1 Introduction

Human activities are increasingly altering ecosystems across the globe, with consequences for both biodiversity and organismal health (Chen et al. 2024; Verma and Prakash 2022). Among these consequences are rising cancer incidence (Dujon, Ujvari, et al. 2024; Giraudeau et al. 2018; Sepp et al. 2019) and the increased spread of symbionts through dispersal and spillover events (Becker et al. 2015; Bullock et al. 2018; Gottdenker et al. 2014). While these two phenomena are typically studied independently, growing evidence suggests they may be closely linked. Tumors can alter host physiology and life‐history traits in ways that increase susceptibility to colonization by opportunistic symbionts (Dujon et al. 2021; Vittecoq et al. 2013; Wang et al. 2017), while some symbionts, particularly parasites and pathogens, can modulate oncogenesis by influencing tumor initiation and progression or by altering host life‐history strategies (Fonti et al. 2023; Michalakis and Hochberg 1994; Thomas et al. 2000). Although the complex and reciprocal links between parasitic and cancer dynamics are increasingly studied, we still know little about how commensals may interact with tumor development, especially given that some can shift toward parasitism under specific conditions (Drew et al. 2021; Maier et al. 2024; Proctor et al. 2023; Stoy et al. 2023). As environmental changes increasingly impact both tumor susceptibility and host‐symbiont associations, there is a growing need for integrative frameworks that explore how these processes influence one another following human‐driven disruptions, an area that remains largely understudied.

In some cases, these disruptions are not permanent: Humans can reintroduce symbiont species into host populations from which they have long been absent, with potentially important ecological and evolutionary consequences. In many instances, including the one considered in this study (see below), it is uncertain whether the host population historically carried the symbiont or retained co‐adapted traits after its loss, making these scenarios closer to an introduction into a naïve system. For example, the arrival of North American gray squirrels ( *Sciurus carolinensis* ) in the United Kingdom brought parapoxviruses into contact with native red squirrels ( *Sciurus vulgaris* ), causing severe mortality, including in regions where the pathogen had been absent for decades (Tompkins et al. 2003). Yet how such (re)introductions might interact with tumor development and progression in wildlife remains largely unknown.

The freshwater cnidarian *Hydra*, and in particular *Hydra oligactis* , is a simple basal metazoan capable of both sexual and asexual reproduction (Yoshida et al. 2006). Under laboratory conditions, some strains can develop tumors, some of which may become transmissible through asexual budding (Boutry et al. 2023; Domazet‐Lošo et al. 2014; Dujon, Boutry, et al. 2024; Tissot, Meliani, Boutry, et al. 2024). Tumoral *H. oligactis* laboratory strains, such as the Saint Petersburg tumorous transmissible line (maintained asexually in culture for over 15 years; Domazet‐Lošo et al. 2014), feature visible tumefactions that typically develop within 3 to 5 weeks following bud emergence (Boutry, Tissot, et al. 2022; Stepanskyy et al. 2025; Tissot et al. 2023), as well as an increased number of tentacles, which have been suggested to result from tumor‐induced manipulation of host phenotype (Boutry, Mistral, et al. 2022; Boutry et al. 2025; Dujon, Boutry, et al. 2024).

In the wild, *Hydra* is frequently colonized by the ciliate *Kerona pediculus* (hereafter referred to as ciliates), a species that lives on the host's external surface (i.e., the ectoderm), feeding on suspended particles or surface debris, and is typically described as commensal (Warren and Robson 1998). Notably, Coleman (1966) reported that ciliate persistence varies with host feeding condition and that ciliates may rely on host‐derived secretions, describing the association as potentially parasitic. To date, the net fitness consequences of this association for the host have not been clearly established, leaving its ecological status ambiguous and potentially dependent on the ecological and physiological context. Some laboratory *Hydra* lines, by contrast, have been maintained ciliate‐free for over 15 years, providing a contrasting context for examining the effects of long‐term symbiont absence. More recently, studies have shown that when given a choice between a healthy and a tumorous hydra, ciliates preferentially colonize tumorous individuals, where they replicate more rapidly and reach higher population densities (Boutry, Mistral, et al. 2022). As a result, tumorous hydras can act as super‐spreaders, facilitating ciliate transmission both within and between host species, and amplifying their populations (Tissot, Meliani, Chee, et al. 2024).

Altogether, the *Hydra*‐ciliate system, with its biological simplicity, experimental accessibility, and the ecological ambiguity of its symbiont, offers a rare opportunity to investigate the interplay between tumor development and symbiosis, particularly along the commensal‐parasite continuum. Yet no study has experimentally tested how long‐term symbiont absence followed by reintroduction interacts with tumor dynamics and host life‐history traits in *Hydra*. This is especially relevant in a context where human‐driven environmental changes are expected to increase cancer susceptibility, disrupt long‐standing associations between hosts and their symbionts, and in some cases reintroduce previously co‐evolved partners into naïve hosts, where the effects can be particularly important under tumor‐prone conditions.

To experimentally investigate these interactions in the context of symbiont reintroduction, we assessed how ciliate re‐exposure, in hosts with different baseline susceptibility to tumors, (i) influences tumor onset, progression, and supernumerary tentacle development, and (ii) affects key host life‐history traits, including age at first reproduction and budding rate. We used two *H. oligactis* lines that have been maintained ciliate‐free in the laboratory for over 15 years: a tumorous line bearing a transmissible tumor (Domazet‐Lošo et al. 2014), and a control line with historically low rates of spontaneous tumor formation under standard laboratory conditions (Boutry, Tissot, et al. 2022). However, during the experiment, individuals from the control line unexpectedly developed tumors at higher frequencies than previously observed, reshaping the experimental framework and enabling comparisons of how symbiont reintroduction affects hosts with either transmissible or *de novo* (spontaneous) tumors.

## 2 Material and Methods

### 2.1 Animals and Culture Conditions

Experiments were conducted using the transmissible tumor lines (TL) and the nontransmissible control lines (CL) of the *H. oligactis* Saint Petersburg strain, both derived from the clonal lineage described in Domazet‐Lošo et al. 2014. Both F0 TL and CL had been maintained under ciliate‐free conditions for over 15 years and were cultivated in mass cultures at 18°C under a 12‐h photoperiod, using Volvic water (a natural spring water routinely used in *Hydra* research for its mineral stability; see Boutry, Tissot, et al. 2022 for rearing protocols). F0 hydras were fed five times per week with *Artemia salina* nauplii to accelerate their asexual reproduction (Tökölyi et al. 2016). The ciliates originated from a separate mass culture of wild *H. oligactis* collected from the field (Montaud lake in France; 43°44′52′′ N; 3°59′23′′ E). For the experiment (see protocol below), F0 hydras from both the TL and the tumor‐free CL were isolated from mass cultures and individually placed into wells of cell culture plates (12‐well cell culture plates; 1.5 mL per well; Thermo Scientific). Following at least 7 days of acclimation, their F1 descendants were then isolated and maintained under a standard feeding regime (three times per week). To avoid artificially amplifying ciliate numbers in the culture plates, the standard water‐change protocol (with water changed 8 h after feeding) was modified by changing the water 1 h after feeding. This timing allowed for the removal of *Artemia* nauplii that had not been ingested by the hydras before they began to decompose, thereby limiting artificial ciliate proliferation and preventing the loss of ciliates that temporarily detach from the host to feed on decaying *Artemia* (preliminary observations, data not shown). A preliminary test confirmed that changing the water at this time point did not significantly reduce ciliate numbers (see Supporting Information). A subsequent cleaning step was deemed unnecessary, as only *Artemia* cuticles remained in the medium. Unlike decomposing nauplii, ciliates were not observed on these cuticles, suggesting they are unlikely to serve as a major resource for ciliate growth, and the cuticles were subsequently removed during the next scheduled water change.

### 2.2 Experimental Protocol

**FIGURE 1:**
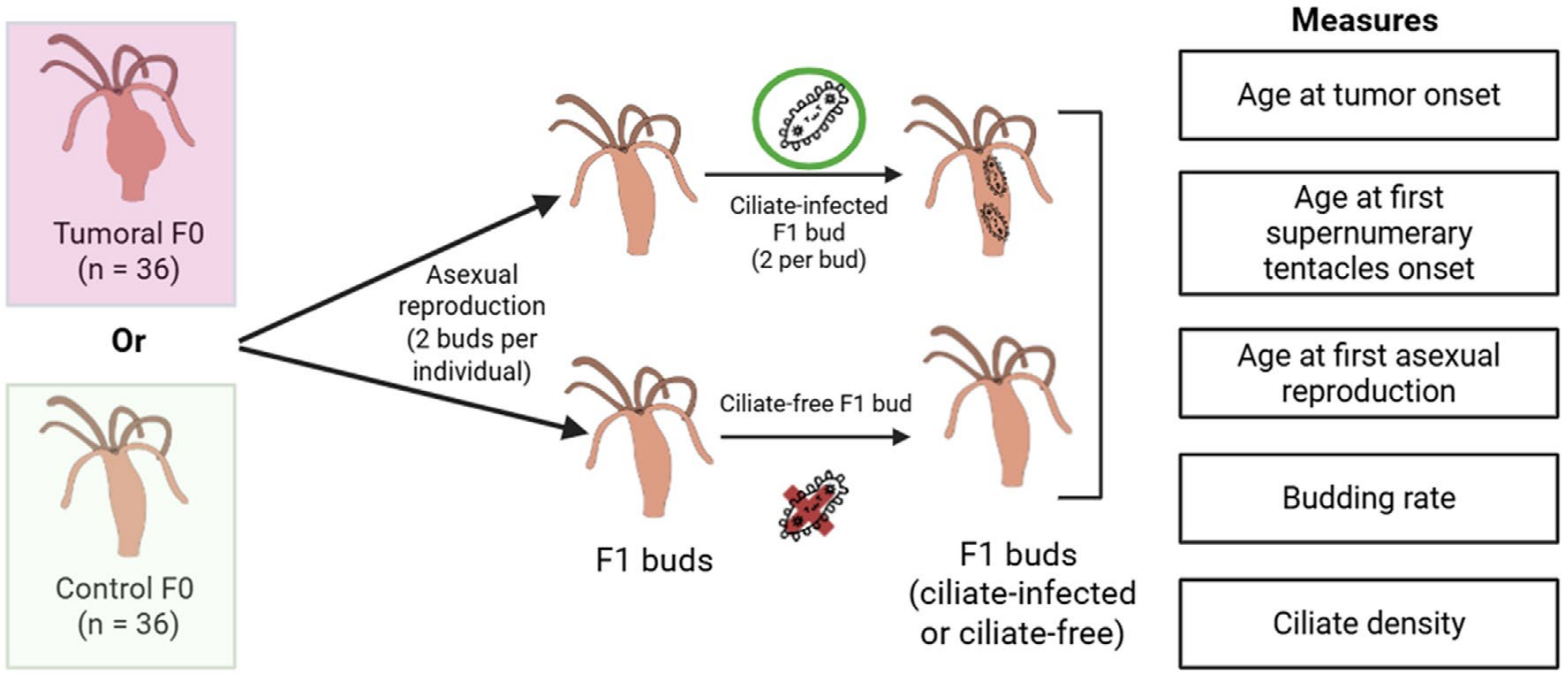
Graphical summary of general experimental design.

A graphical summary of the experimental design is presented in Figure 1. For the experiment, 36 TL and 36 CL ciliate‐free *H. oligactis* from the St. Petersburg strain were individually isolated to constitute the F0 generation (sample size based on logistical feasibility and previous studies showing sufficient statistical power; e.g., Boutry, Tissot, et al. 2022). Tumor phenotype intensity of F0 tumorous individuals was recorded using the six‐level visual scale described in Tissot et al. (2023), which combines two criteria characteristics of the TL: the extent of body deformation and the presence of supernumerary tentacles. Scores range from 0 (no visible symptoms) to 5 (severe body deformation with supernumerary tentacles). Each F0 individual was expected to produce two F1 buds within a week, which were collected at the beginning of the week; their emergence dates (hereafter referred to as births or birth dates) were recorded. Individuals that produced only one bud during a week were excluded from the weekly sampling, and collection was postponed to the following week. In total, 144 F1 buds were expected. Small batches (e.g., fewer than three F1 pairs) were excluded, and the procedure was repeated the following week if necessary. Potential batch effects arising from these weekly cohorts were accounted for in the analyses (see Data analysis section).

F1 buds were then individually placed into wells of 12‐well plates (1.5 mL/well) and randomly assigned to infected or noninfected treatment groups, to minimize potential experimenter bias. This randomization was stratified by parental line to ensure balanced representation of each F0 parent across treatments and experimental batches. In the infected group, two ciliates were introduced per well to increase the likelihood of successful infection, following a similar approach described by Tissot, Meliani, Chee, et al. (2024). These were aspirated along with 5 μL of water from a mass culture of ciliates maintained on field‐derived *H. oligactis* , using a P10 pipette (Thermo Finnpipette) under a dissecting microscope. The 5 μL solution, containing two ciliates, was then transferred into a well containing an F1 hydra. In the noninfected group, 5 μL of water from the same ciliate culture (without ciliates) was introduced. The presence of ciliates was verified 15 h post‐infestation, prior to the onset of asexual reproduction. The two‐ciliate infection method demonstrated high success, as only one F1 hydra lost its ciliates within the first 7 days post‐infestation; this individual was subsequently excluded from the analysis.

Three times a week, before feeding and until the fifth month of the experiment (a duration limited by logistical constraints), we recorded the following variables: age at first reproduction, age at first supernumerary tentacle development (defined as the appearance of the eighth tentacle), age at tumor onset (defined as the appearance of visible tumors on the main body of the hydra), budding rate, and ciliate density. Hydras from CL that developed tumors spontaneously during the experiment were referred to as STH, while those from TL that developed visible tumors were referred to as TTH. Ciliate density was categorized into five classes, based on preliminary observations (data not shown) of typical abundance and the ease and accuracy with which individuals could be counted on infected hydras. The five classes of density were defined as follows: 1–9 ciliates (Class A), 10–19 (Class B), 20–29 (Class C), 30, or more (Class D), and those who lost their ciliates (Class 0), with representative examples provided in the Supporting Information. Buds were removed from individual wells after being counted, and the number of ciliates present on them was recorded prior to removal. Raw ciliate counts observed on buds are provided in the Supporting Information.

### 2.3 Data Analysis

All data processing and statistical analyses were performed using R software (version 4.2.2; R Core Team 2022) within the RStudio integrated development environment (Posit team 2025). Generalized linear mixed‐effects models (GLMMs) were fitted using the “glmmTMB” package (Brooks et al. 2017) when appropriate. Model selection followed the three‐step procedure described in Zuur et al. (2009): first, we selected the random effect structure (including F0 identity, batch, F0 tumor intensity, and F1 identity where appropriate; see below for details) using the full model fitted with restricted maximum likelihood (REML; see Millar 2011 for non‐Gaussian distributions); second, we determined the fixed effect structure using maximum likelihood (ML); and third, we refitted the selected model using REML. To identify the most parsimonious model at each step, model selection was based on the corrected Akaike Information Criterion (AICc) and associated AICc weight, calculated using the MuMIn package (Bartoń 2023). The fit of the data to the model was evaluated using the DHARMa package (Hartig 2022) through examination of residual distribution, including Kolmogorov–Smirnov tests, overdispersion diagnostics and outlier tests. When applicable, pairwise comparisons were made using estimated marginal means (via the “emmeans” package), with *p*‐values adjusted for multiple comparisons using the Benjamini–Hochberg correction (Lenth et al. 2023). A full list of additional R packages used is provided in the Supporting Information. Ciliate density, recorded as a five‐level categorical variable (see section above), was included only in the analysis of budding rate. For traits such as age at first reproduction, tumor onset, and supernumerary tentacle development, the most frequent density class observed during the period preceding the event was excluded from analysis due to insufficient and unbalanced sample sizes across classes. Similarly, because only a small number of individuals in both lineages did not develop tumors (see the Results section), these were excluded from all analyses except for tumor onset, where including both tumorous and nontumorous individuals was necessary to assess incidence. This exclusion helped avoid extreme data imbalance (e.g., groups where nearly all individuals developed tumors), which would lead to limited statistical power and numerical instability in model estimation.

#### 2.3.1 Effects of Ciliate Infection on Tumor and Supernumerary Tentacle Development

This analysis aimed to evaluate whether ciliate infection affects the probability and timing of tumor onset and supernumerary tentacle development. All GLMMs included F0 identity, batch, and F0 tumor intensity as random intercepts to account for nonindependence due to repeated measures and batch‐related variability. F0 tumor intensity was also included as a categorical random intercept to account for potential shared variance among F1 offspring originating from parents with the same disease severity level. The probability of tumor onset and supernumerary tentacle development was modeled using binomial GLMMs, with lineage (TL or CL) and infection status (infected or uninfected) as fixed effects. The timing of tumor onset and supernumerary tentacle development was modeled using negative binomial GLMMs, with the same fixed effects as in the binomial model, except that only individuals who developed tumors (TTH and STH; i.e., differing by tumor context) were included.

#### 2.3.2 Effects of Ciliate Infection on Life‐History Traits

This analysis aimed to evaluate whether ciliate infection influences key life‐history traits, including age at first asexual reproduction and budding rate.

##### 2.3.2.1 Asexual Reproduction

We modeled the effects of ciliate infection on age at first reproduction using a Poisson GLMM. To assess weekly budding rate, we fitted two separate Poisson models: one testing the effect of ciliate infection (infected vs. uninfected), and another testing the effect of ciliate density (within the infected group, classified into five levels). Ciliate density was determined weekly for each F1 individual as the most frequent class to align the predictor with the weekly scale of the budding rate and to represent the sustained ciliate exposure during that period. All models included tumor context (TTH or STH) as a fixed effect, along with the same random effects structure as described above (F0 identity, batch, and F0 tumor intensity). Infection status (infected vs. not infected) was included as a fixed effect in all models except the ciliate density budding model, where only infected individuals were considered. In the budding rate models, week (F1 age, in weeks) was also included as a fixed effect to account for temporal variation in reproduction, and F1 identity was added as a random intercept to account for repeated measures within individuals.

## 3 Results

### 3.1 Effects of Ciliate Infection on Tumor and Supernumerary Tentacle Development

#### 3.1.1 Tumor Onset and Timing

**FIGURE 2:**
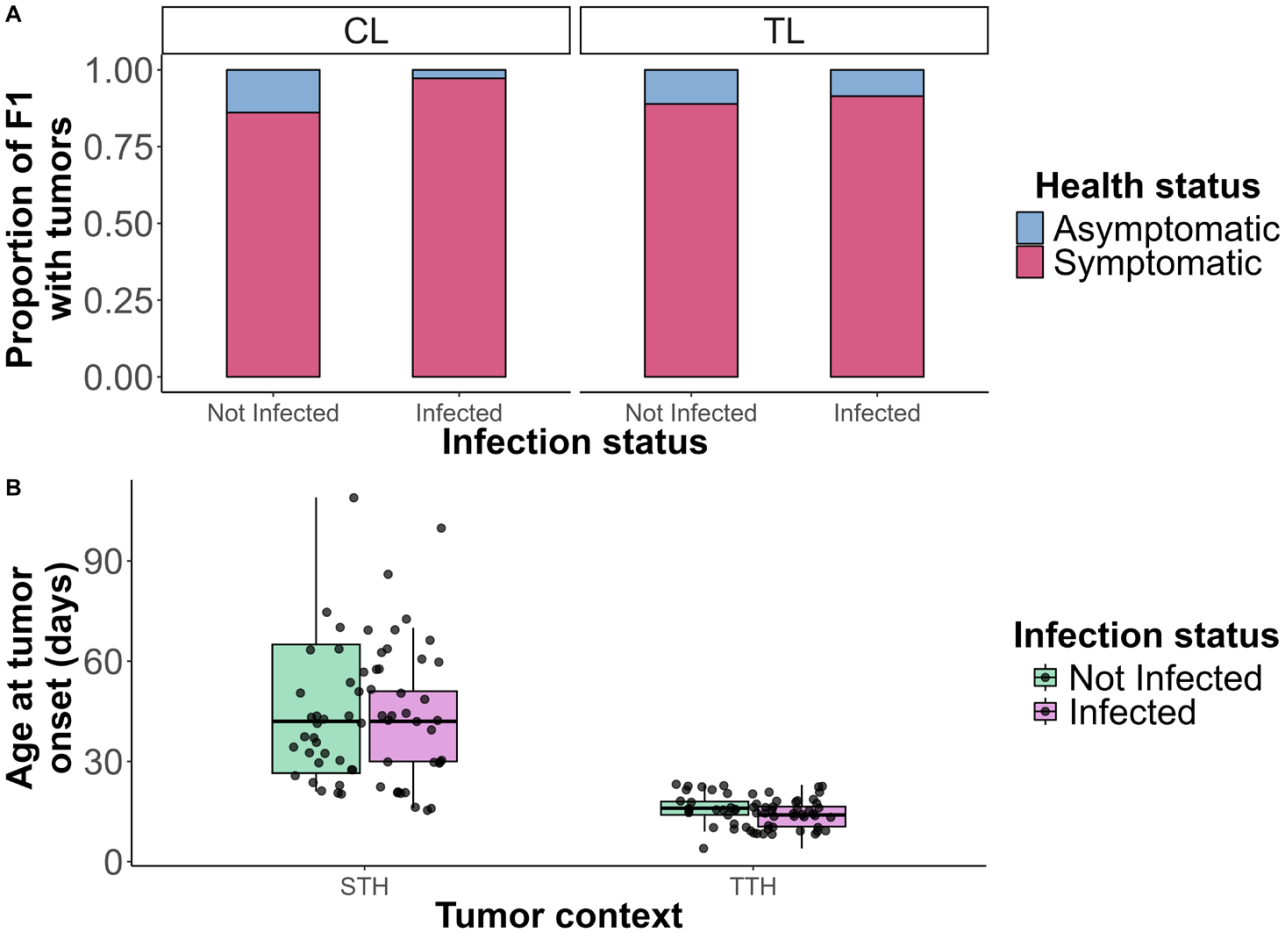
Tumor incidence and onset timing. (A) Bar plots show the proportion of F1 that became symptomatic across infection status (not infected or infected) and lineage (transmissible tumor line, TL; control line, CL). Asymptomatic F1 are shown in blue, and symptomatic F1 in pink. (B) Boxplots show the age at tumor onset (in days) across tumor contexts (spontaneous tumoral hydras, STH; transmissible tumoral hydras, TTH), for both infected (purple) and not infected (green) F1.

Ciliate infection did not significantly affect the likelihood of tumor development (Figure 2A; binomial GLMM; *p* = 0.102). As expected, most individuals from TL developed tumors, with 32 out of 36 (89%) in the noninfected group and 33 out of 35 (94%) in the infected group. A similarly high proportion of CL individuals developed spontaneous tumors, with 31 out of 36 in the noninfected group and 35 out of 36 in the infected group. Accordingly, lineage had no significant effect on tumor incidence, as it was not retained in the best‐fitting model. Although this high incidence in the CL lineage precluded comparisons with a truly tumor‐free control group, thereby limiting our ability to assess infection effects in healthy hosts, it revealed a striking shift in tumor susceptibility, enabling comparisons of how symbiont reintroduction affects hosts with either transmissible (TTH) or *de novo* (spontaneous, STH) tumor contexts. While infection did not alter overall tumor incidence, it accelerated tumor onset across tumor contexts by 13% (Figure 2B; negative binomial GLMM; IRR = 0.86, CI [0.77–0.96], *p* = 0.008). This corresponded to model‐predicted tumor onset occurring approximately 6 days earlier in the STH and 2 days earlier in the TTH. In addition, tumors appeared significantly earlier in TTH than in STH, with a 63% shorter delay on average (Figure 2B; negative binomial GLMM; IRR = 0.37, CI [0.31–0.44], *p* < 0.001), corresponding to a model‐predicted difference of approximately 29 days (46 vs. 17 days).

#### 3.1.2 Supernumerary Tentacles Onset and Timing

**FIGURE 3:**
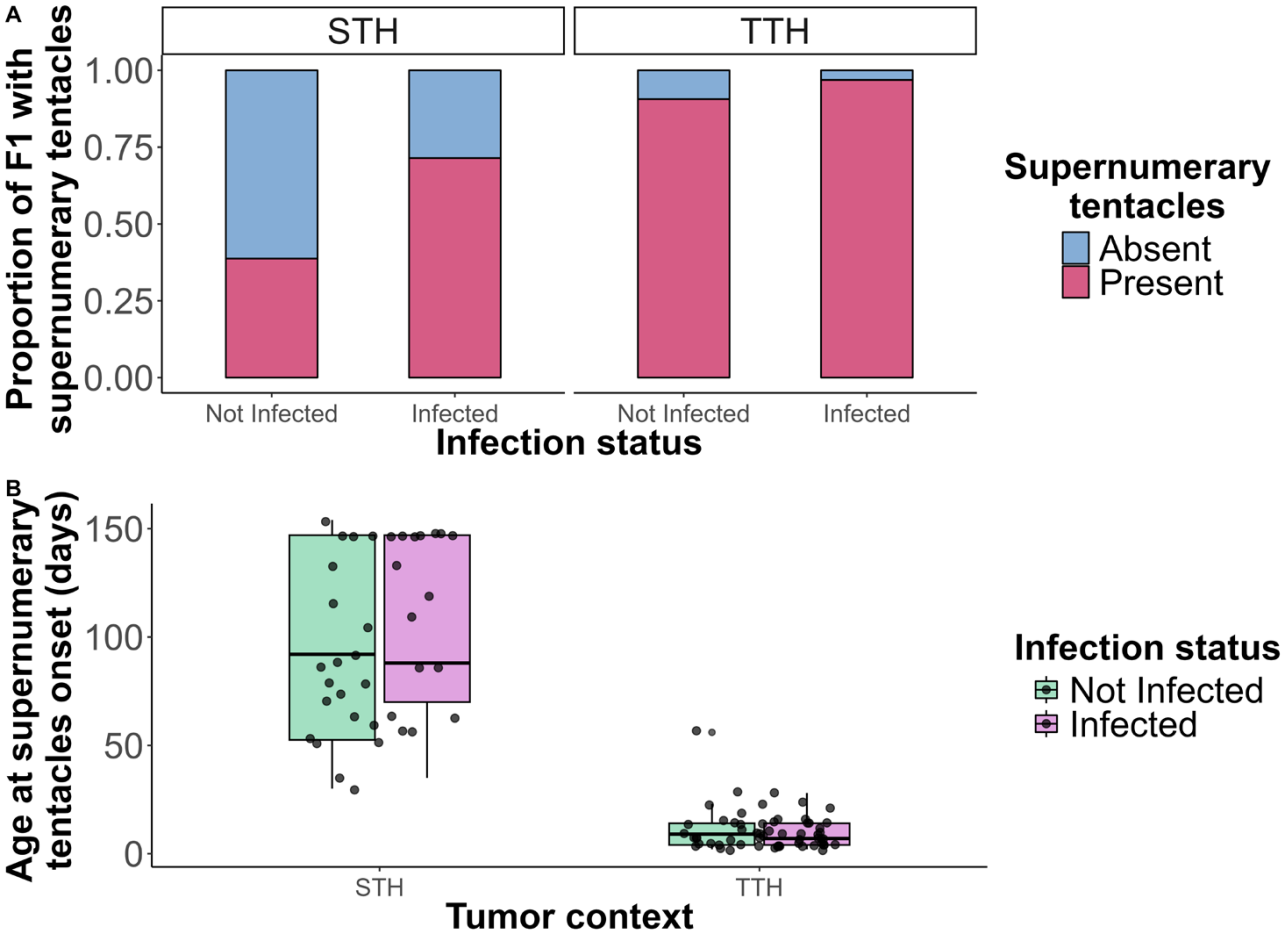
Supernumerary tentacles incidence and onset timing in tumor‐bearing hydras. (A) Bar plots show the proportion of F1 developing supernumerary tentacles (≥ 8) across infection status (not infected or infected) and tumor contexts (transmissible tumoral hydras, TTH; spontaneous tumoral hydras, STH). F1 without supernumerary tentacles are shown in blue, and those with supernumerary tentacles in pink. (B) Boxplots show the age at first appearance of supernumerary tentacles (in days) across tumor contexts (TTH vs. STH), for both infected (purple) and not infected (green) F1.

The probability of developing supernumerary tentacles increased with both ciliate infection and tumor context. Infected individuals were 5.5 times more likely to develop supernumerary tentacles (Figure 3A; binomial GLMM; OR = 5.49, CI [1.23–24.49], *p* = 0.026), and TTH were 17.8 times more likely compared to STH (Figure 3A; binomial GLMM; OR = 17.77, CI [2.99–105.45], *p* = 0.002). Although the near‐complete separation of supernumerary tentacles in certain groups results in wide confidence intervals and affects the precision of the OR estimates, both effects remain statistically significant. However, infection did not significantly affect the timing of supernumerary tentacle emergence (the best‐fitting model did not retain the infection variable). In contrast, TTH developed supernumerary tentacles significantly earlier than STH, with an 89% shorter delay on average (Figure 3B; negative binomial GLMM; IRR = 0.11, CI [0.09–0.14], *p* < 0.001), corresponding to a model‐predicted difference of approximately 87 days (98 vs. 11 days).

### 3.2 Effects of Ciliate Infection on Life‐History Traits

#### 3.2.1 Age at First Asexual Reproduction

**FIGURE 4:**
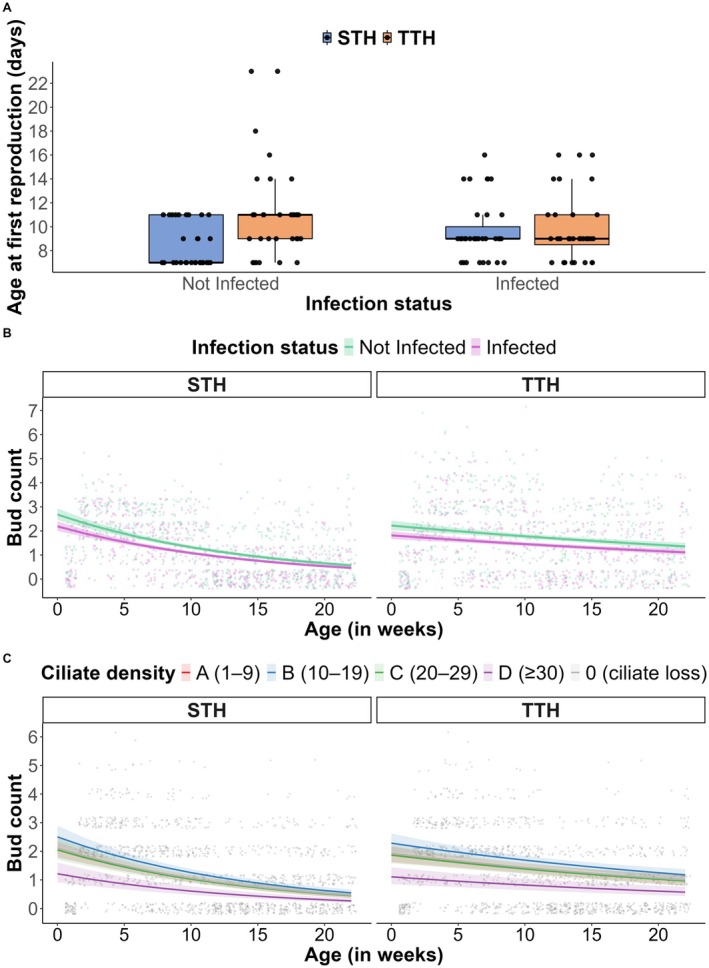
Asexual reproduction traits in tumor‐bearing hydras. (A) Boxplots show the age at first reproduction (in days) across tumor contexts (spontaneous tumoral hydras, STH; transmissible tumoral hydras, TTH) and infection statuses (not infected or infected). STH are shown in blue and TTH in orange. (B, C) Scatter plots show weekly budding rate across tumor contexts (STH vs. TTH), depending on infection status (Panel B; green for not infected and purple for infected) or ciliate density among infected individuals (Panel C; red for A, blue for B, green for C, purple for D, and gray for 0). Lines represent model‐predicted average values with 95% confidence intervals.

The effect of tumor context on age at first reproduction varied by infection status. In the absence of infection, TTH reproduced 32% later than STH (Figure 4A; Poisson GLMM; IRR = 1.32, 95% CI = [1.07–1.64], *p* = 0.003), corresponding to model‐predicted averages of approximately 11.2 and 8.4 days, respectively. This difference was no longer apparent under infection, where TTH and STH reproduced at similar times (9.9 days for TTH vs. 9.6 days for STH, *p* = 0.75). This apparent convergence resulted from opposing nonsignificant trends: infection was associated with a slight delay in STH (from 8.4 to 9.6 days, *p* = 0.14) and a slight acceleration in TTH (from 11.2 to 9.9 days, *p* = 0.14). While neither trend was statistically significant within tumor contexts, their combination resulted in a lack of detectable difference in age at first reproduction between TTH and STH observed under infected conditions.

#### 3.2.2 Asexual Reproduction Rate

Ciliate infection had a significant negative effect on asexual reproduction. Infected individuals produced 17% fewer buds per week than not infected ones, regardless of tumor context (Figure 4B; Poisson GLMM; IRR = 0.83, 95% CI = [0.74–0.93], *p* = 0.002). Budding rate also declined over time in all groups (Figure 4B; Poisson GLMM; IRR = 0.93, 95% CI = [0.92–0.94], *p* < 0.001). TTH initially reproduced less than STH (Figure 4B; Poisson GLMM; IRR = 0.80, 95% CI = [0.65–0.99], *p* = 0.037), but the decline in budding rate over time was less pronounced in the TTH (Figure 4B; Poisson GLMM; interaction IRR = 1.04, 95% CI = [1.03–1.05], *p* < 0.001), suggesting that while TTH started with lower budding activity, they maintained it better over time.

Within infected hydras, budding rate was lowest in Class D (≥ 30 ciliates), which performed significantly worse than Class B (Figure 4C; Poisson GLMM; IRR = 0.57, 95% CI [0.39–0.83], *p* < 0.001), Class C (Figure 4C; Poisson GLMM; IRR = 0.66, 95% CI [0.44–0.98], *p* = 0.01), and Class 0 (Figure 4C; Poisson GLMM; IRR = 0.67, 95% CI [0.44–1.03], *p* = 0.02), and showed a marginal trend toward lower budding than Class A (Figure 4C; Poisson GLMM; IRR = 0.74, 95% CI [0.50–1.09], *p* = 0.06). Conversely, Class B (10–19 ciliates) exhibited the highest budding rate, significantly outperforming Class A (Figure 4C; Poisson GLMM; IRR = 1.31, 95% CI [1.08–1.60], *p* = 0.0004) and Class D, and showing marginal trends toward higher rates than Class C (Figure 4C; Poisson GLMM; IRR = 1.16, 95% CI [0.93–1.45], *p* = 0.09) and Class 0 (Figure 4C; Poisson GLMM; IRR = 1.19, 95% CI [0.92–1.54], p = 0.09). Budding rates among Classes 0, A, and C did not differ significantly. Although sample sizes across density classes were not balanced, these results suggest that, within infected individuals, moderate ciliate densities appear to be associated with increased asexual reproduction, whereas high densities appear detrimental.

## 4 Discussion

In a context where environmental changes increasingly disrupt host–symbiont relationships and increase tumor susceptibility, the *Hydra*‐ciliate system ( *H. oligactis* and its ectosymbiotic ciliate *K. pediculus*) offers valuable opportunity to examine the interplay between tumor development and symbiont reintroduction in shaping tumor dynamics, physiology, and life‐history traits in hosts with different baseline susceptibility to tumors. As outlined in previous sections, the unexpectedly high incidence of spontaneous tumors in CL precluded comparisons with a truly tumor‐free control group, thereby limiting our ability to assess infection effects in healthy hosts. While this reshaped our experimental framework, rather than compromising the study, it allowed for a unique comparison of how symbiont reintroduction affects hosts within either transmissible or *de novo* (spontaneous) tumor contexts. We therefore discuss our findings in light of both the intrinsic differences between spontaneous and transmissible tumors, and how these tumor contexts are influenced by ciliate infection across key aspects of host biology.

### 4.1 Unexpected Incidence of Spontaneous Tumors

One of the unexpected results of this study was the high incidence of spontaneous tumor development in CL. Although rare spontaneous tumors have been observed in this strain in the past, their frequency in this experiment markedly exceeded previously reported levels under standard laboratory conditions (Boutry, Mistral, et al. 2022; Boutry, Tissot, et al. 2022). Several nonexclusive hypotheses could explain this outcome, although they require further experimental confirmation. First, although CL has been maintained free of transmissible tumors for over 15 years, it shares the same genetic origin as TL (despite some possible somatic mutations). It is therefore possible that latent tumorigenic potential remains in the genome and may re‐emerge under certain unknown conditions. Indeed, the original TL itself emerged from a hydra with spontaneous tumors in the same strain (Domazet‐Lošo et al. 2014), raising the hypothesis that we could be witnessing the early stages of a new transmissible tumor line, although there is currently no direct evidence for this in the present study. Second, although noninfected individuals were not exposed to ciliates, they did receive inoculum water that had previously been in contact with both ciliates and tumorous hydras. While this solution was filtered to remove ciliates, it may still have contained soluble tumorigenic factors, including microbiota capable of promoting tumor formation in predisposed individuals. Tissot et al. (2023) using the same CL reported similarly high rates of spontaneous tumors under a different feeding regime, where hydras were fed more frequently (five times per week) than in the present study (three times per week). The authors proposed several possibilities, including that altered food availability could affect microbiome composition and thereby affect tumorigenesis (Tissot et al. 2023). This possibility remains actively debated in the *H. oligactis* literature (Boutry et al. 2023; Rathje et al. 2020; Stepanskyy et al. 2025; Tissot, Meliani, Boutry, et al. 2024) and is increasingly supported by findings from other systems linking microbiota to cancer susceptibility (Fulbright et al. 2017; Garrett 2015). In this context, it remains to be confirmed whether the spontaneous tumors observed in CL share microbial, but also cellular and molecular features with those previously reported. Furthermore, while the precise cause of the unexpectedly high tumor incidence in CL remains unclear, the potential role of filtered inoculum water represents a limitation of this study, as it could have acted as a confounding factor across all groups. Replicating the design using inoculum water alone could help determine whether soluble factors promote tumor formation, and testing *Hydra* strains with no documented tumor cases may clarify whether this effect reflects a broader susceptibility to microbiota‐driven dysbiosis.

### 4.2 Differences Between Spontaneous and Transmissible Tumors

Despite sharing a common genetic background, STH and TTH differed in tumor dynamics and reproductive traits, confirming that tumor context and long‐term coevolutionary dynamics strongly shape host–tumor interactions. Tumors in TTH developed earlier and were more frequently associated with supernumerary tentacles, hallmarks of the long‐maintained transmissible line and likely reflect coevolution between tumor cells and their host (Boutry, Mistral, et al. 2022; Boutry, Tissot, et al. 2022; Stepanskyy et al. 2025). In contrast, tumors in STH appeared later and were less often accompanied by supernumerary tentacles, consistent with the idea that these newly arisen tumors have not yet undergone similar evolutionary refinement. Indeed, spontaneous tumors arising in CL are intentionally eliminated early under laboratory conditions, limiting their opportunity for persistence or adaptation. This difference in maintenance history may help explain the greater variability observed in STH; while TTH derive from a transmissible tumor lineage shaped by long‐term propagation and repeated artificial selection, STH represent sporadic cases that have not undergone similar selective processes and may therefore reflect a wider range of spontaneous phenotypes. However, given that tumor transmissibility can evolve within a few generations (Tissot, Meliani, Boutry, et al. 2024), it is plausible that similar features could emerge in STH if allowed to persist.

These divergent tumor dynamics may help explain differences in reproductive patterns. STH reproduced earlier and more intensely at first, but their budding rate declined more sharply over time. TTH, by contrast, reproduced later and more slowly at first, but maintained a steadier budding rate across the study period. Several hypotheses could account for the pattern, although causal inference is limited in the absence of direct testing. One possibility is that STH reflect a baseline reproductive pattern typical of tumor‐free individuals, and that the later emergence of tumors disrupts reproduction only at later stages, causing a decline. However, this view contrasts with prior work showing that TTH tend to reproduce more intensely early in life as a compensatory response to tumor burden (Boutry, Tissot, et al. 2022). If STH were merely tumor‐free early on, their initial reproduction would likely be lower, not higher, than that of TTH. This suggests that STH may also engage in a compensatory reproductive strategy, possibly in a more pronounced manner. However, because STH represent newly emerged tumors without a long‐term association with their host, this pattern is more likely to reflect a generalized physiological response to the early presence of tumor cells, rather than a specific reproductive strategy shaped by long‐term association with tumors. Alternatively, we hypothesize that the high budding rate in STH (e.g., due to unknown physiological responses to inoculum exposure) could potentially contribute to tumor development, as budding involves intense cell proliferation that may promote dysregulation in genetically predisposed individuals. This would be consistent with classical life‐history trade‐offs, in which elevated reproductive effort compromises somatic maintenance and increase vulnerability to cancer (Jacqueline et al. 2016). In contrast, TTH individuals already harbor a long‐established transmissible tumor lineage, and their reproductive dynamics likely reflect physiological responses to the persistent presence of tumor cells rather than a cause of tumor emergence. A third possibility is that the transmissible tumor lineage could be evolving toward earlier tumor onset, potentially reducing the window for early‐life reproduction. This could explain why TTH do not show the same initial reproductive intensity as STH.

Altogether, our findings underscore that the evolutionary context of tumors, whether newly emerged or long established, can profoundly shape host reproductive allocation. These results highlight the importance of considering not only tumor presence but also its temporal dynamics and evolutionary background when evaluating host life‐history responses.

### 4.3 Ciliate Infection Across Tumor Contexts

Ciliate infection influenced multiple aspects of host biology, including reproduction, tumor progression, and supernumerary tentacle development, with no strong evidence for differences between tumor contexts. Overall, infected hydras produced fewer buds than uninfected ones, with the reduction becoming more pronounced at high ciliate densities. In parallel, infection appeared to attenuate the difference in age at first reproduction previously observed between uninfected TTH and STH, although the opposing trends within each tumor context lacked statistical significance, likely due to limited power. Infection was also associated with earlier tumor onset, and a higher probability of supernumerary tentacle formation. Several nonexclusive hypotheses could explain these effects.

Although ciliates remain external, their presence and feeding behavior may alter the host's epithelial surface microenvironment. In *Hydra*, epithelial homeostasis is tightly coupled to tissue regeneration and budding, and depends on interactions between epithelial and interstitial cells (Shostak 2017). Disruption of this interface could reduce budding efficiency, especially at high symbiont densities, as observed in individuals with more than 30 ciliates. By perturbing epithelial homeostasis, ciliate infection may also disrupt morphogenetic patterning, potentially contributing to the increased frequency of supernumerary tentacles observed in infected hydras. This could reflect a disruption of the epithelial signaling processes that govern tentacle morphogenesis in *Hydra* (Vogg et al. 2022), or alternatively, a compensatory response to infection‐driven stress. Indeed, hydras experiencing reduced budding might invest in tentacle growth to enhance prey capture (Boutry, Mistral, et al. 2022), a plastic strategy that could help restore reproductive output since resource intake is positively linked to budding (Tökölyi et al. 2016), although the underlying mechanisms remain to be elucidated.

In addition to direct effects on epithelial homeostasis, ciliate infection might indirectly influence host physiology by introducing microbial associates or altering the host's native microbiota. While protozoa have been shown to act as microbial vectors in other systems (Balczun and Scheid 2017; Margarita et al. 2020), the extent to which this occurs in our study remains to be determined as microbial composition was not assessed. This perspective is particularly relevant since the *Hydra* microbiome is known to modulate various aspects of host physiology (He and Bosch 2022; Murillo‐Rincon et al. 2017), and is frequently associated with the presence of tumors in *H. oligactis* (Boutry et al. 2023; Rathje et al. 2020; Stepanskyy et al. 2025). Future work should therefore investigate whether ciliate colonization leads to shifts in the microbiota, and whether such shifts contribute to the physiological patterns observed here.

Another factor worth considering is the evolutionary history between host and symbiont. The *Hydra* lines used in this study have been maintained in the laboratory without exposure to *K. pediculus* for over 15 years, which corresponds to approximately 500–700 generations, assuming a constant generation time of 8 to 11 days. From an evolutionary perspective, this prolonged absence may have disrupted co‐adaptative dynamics that previously existed between host and symbiont. While this hypothesis remains to be tested, it is possible that these *Hydra* may now be naïve to *K. pediculus*, lacking the regulatory or tolerance mechanisms needed to buffer its physiological effects. Such a breakdown in host‐symbiont coadaptation could amplify infection‐induced changes by increasing host sensitivity or reducing the host's ability to regulate or tolerate the symbiont. As an illustrative analogy, similar phenomena have been observed in other systems: for instance, sigma virus infections in *Drosophila* show that host shifts into evolutionarily naïve species can dramatically increase viral virulence (Longdon et al. 2011, 2015), highlighting how disrupted coevolutionary history can intensify physiological responses. Lastly, as outlined earlier in the Introduction, it remains uncertain whether this specific host population historically carried the symbiont, a scenario that could also reflect an introduction into a naïve host.

## 5 Conclusion

Together, our findings reveal that both symbiont reintroduction and tumor context can reshape tumor dynamics and host life‐history traits in tumor‐bearing *H. oligactis* . This work underscores the importance of considering symbiont reintroductions, especially following prolonged absence, in shaping host physiology, tumor dynamics, and life‐history traits in hosts physiologically compromised by internal stressors such as tumors. Furthermore, these findings suggest that *K. pediculus* may not always act as a strict commensal, especially at high densities, highlighting its potential to exert deleterious effects in hosts presumably lacking recent coevolutionary history with the symbiont or already burdened by tumors. More broadly, as environmental changes can disrupt host–symbiont associations and elevate cancer susceptibility, this study provides experimental insight into how such pressures might shape disease dynamics, host fitness, and the ecological roles of reintroduced symbionts in vulnerable host populations.

## Author Contributions

**Nikita Stepanskyy:** conceptualization (equal), data curation (equal), formal analysis (equal), investigation (equal), methodology (equal), visualization (equal), writing – original draft (equal), writing – review and editing (equal). **Jordan Meliani:** resources (equal), writing – review and editing (equal). **Jácint Tökölyi:** validation (equal), writing – review and editing (equal). **Aurora M. Nedelcu:** validation (equal), writing – review and editing (equal). **Beata Ujvari:** validation (equal), writing – review and editing (equal). **Frédéric Thomas:** conceptualization (equal), funding acquisition (equal), supervision (equal), validation (equal), writing – original draft (equal), writing – review and editing (equal). **Antoine M. Dujon:** conceptualization (equal), formal analysis (equal), supervision (equal), validation (equal), visualization (equal), writing – original draft (equal), writing – review and editing (equal).

## Funding

This work was funded by the CNRS, the HOFFMANN Family, and the following grant: ANR EVOSEXCAN (ANR‐23‐CE13‐0007).

## Conflicts of Interest

The authors declare no conflicts of interest.

## Supporting information


**Appendix S1:** ece373461‐sup‐0001‐AppendixS1.zip.

## Data Availability

Scripts, data, and additional files associated with this study are provided in the Supporting Information.

## References

[ece373458-bib-0001] Balczun, C. , and P. L. Scheid . 2017. “Free‐Living Amoebae as Hosts for and Vectors of Intracellular Microorganisms With Public Health Significance.” Viruses 9: 65. 10.3390/v9040065. 28368313 PMC5408671

[ece373458-bib-0002] Bartoń, K. 2023. “MuMIn: Multi‐Model Inference. R package version 1.47.5.” https://CRAN.R‐project.org/package=MuMIn.

[ece373458-bib-0003] Becker, D. J. , D. G. Streicker , and S. Altizer . 2015. “Linking Anthropogenic Resources to Wildlife–Pathogen Dynamics: A Review and Meta‐Analysis.” Ecology Letters 18: 483–495. 10.1111/ele.12428. 25808224 PMC4403965

[ece373458-bib-0004] Boutry, J. , M. Buysse , S. Tissot , et al. 2023. “Spontaneously Occurring Tumors in Different Wild‐Derived Strains of *Hydra* .” Scientific Reports 13: 7449. 10.1038/s41598-023-34656-0. 37156860 PMC10167321

[ece373458-bib-0005] Boutry, J. , J. Mistral , L. Berlioz , et al. 2022. “Tumors (Re)shape Biotic Interactions Within Ecosystems: Experimental Evidence From the Freshwater Cnidarian *Hydra* .” Science of the Total Environment 803: 149923. 10.1016/j.scitotenv.2021.149923. 34487898

[ece373458-bib-0006] Boutry, J. , O. Rieu , L. Guimard , et al. 2025. “First Evidence for the Evolution of Host Manipulation by Tumors During the Long‐term Vertical Transmission of Tumor Cells in *Hydra oligactis* .” eLife 13: RP97271. 10.7554/eLife.97271.3. 40036153 PMC11879105

[ece373458-bib-0007] Boutry, J. , S. Tissot , N. Mekaoui , et al. 2022. “Tumors Alter Life History Traits in the Freshwater Cnidarian, *Hydra oligactis* .” iScience 25: 105034. 10.1016/j.isci.2022.105034. 36147948 PMC9485901

[ece373458-bib-0008] Brooks, M. E. , K. Kristensen , K. J. van Benthem , et al. 2017. “glmmTMB Balances Speed and Flexibility Among Packages for Zero‐Inflated Generalized Linear Mixed Modeling.” R Journal 9, no. 2: 378–400. 10.32614/RJ-2017-066.

[ece373458-bib-0009] Bullock, J. M. , D. Bonte , G. Pufal , et al. 2018. “Human‐Mediated Dispersal and the Rewiring of Spatial Networks.” Trends in Ecology & Evolution 33: 958–970. 10.1016/j.tree.2018.09.008. 30314915

[ece373458-bib-0010] Chen, F. , F. Jiang , J. Ma , M. A. Alghamdi , Y. Zhu , and J. W. H. Yong . 2024. “Intersecting Planetary Health: Exploring the Impacts of Environmental Stressors on Wildlife and Human Health.” Ecotoxicology and Environmental Safety 283: 116848. 10.1016/j.ecoenv.2024.116848. 39116691

[ece373458-bib-0011] Coleman, D. C. 1966. “The Laboratory Population Ecology of *Kerona Pediculus* (O.F.M.) Epizoic on *Hydra* SPP.” Ecology 47: 705–711. 10.2307/1934258.

[ece373458-bib-0012] Domazet‐Lošo, T. , A. Klimovich , B. Anokhin , et al. 2014. “Naturally Occurring Tumours in the Basal Metazoan *Hydra* .” Nature Communications 5: 4222. 10.1038/ncomms5222. 24957317

[ece373458-bib-0013] Drew, G. C. , E. J. Stevens , and K. C. King . 2021. “Microbial Evolution and Transitions Along the Parasite–Mutualist Continuum.” Nature Reviews. Microbiology 19: 623–638. 10.1038/s41579-021-00550-7. 33875863 PMC8054256

[ece373458-bib-0014] Dujon, A. M. , J. Boutry , S. Tissot , et al. 2024. “The Widespread Vulnerability of *Hydra oligactis* to Tumourigenesis Confirms Its Value as a Model for Studying the Effects of Tumoural Processes on the Ecology and Evolution of Species.” Science of the Total Environment 951: 175785. 10.1016/j.scitotenv.2024.175785. 39187082

[ece373458-bib-0015] Dujon, A. M. , J. S. Brown , D. Destoumieux‐Garzón , et al. 2021. “On the Need for Integrating Cancer Into the One Health Perspective.” Evolutionary Applications 14: 2571–2575. 10.1111/eva.13303. 34815739 PMC8591323

[ece373458-bib-0016] Dujon, A. M. , B. Ujvari , S. Tissot , et al. 2024. “The Complex Effects of Modern Oncogenic Environments on the Fitness, Evolution and Conservation of Wildlife Species.” Evolutionary Applications 17: e13763. 10.1111/eva.13763. 39100750 PMC11294924

[ece373458-bib-0017] Fonti, N. , F. Parisi , F. Mancianti , G. Freer , and A. Poli . 2023. “Cancerogenic Parasites in Veterinary Medicine: A Narrative Literature Review.” Infectious Agents and Cancer 18: 45. 10.1186/s13027-023-00522-x. 37496079 PMC10373346

[ece373458-bib-0018] Fulbright, L. E. , M. Ellermann , and J. C. Arthur . 2017. “The Microbiome and the Hallmarks of Cancer.” PLoS Pathogens 13: e1006480. 10.1371/journal.ppat.1006480. 28934351 PMC5608396

[ece373458-bib-0019] Garrett, W. S. 2015. “Cancer and the Microbiota.” Science 348: 80–86. 10.1126/science.aaa4972. 25838377 PMC5535753

[ece373458-bib-0020] Giraudeau, M. , T. Sepp , B. Ujvari , P. W. Ewald , and F. Thomas . 2018. “Human Activities Might Influence Oncogenic Processes in Wild Animal Populations.” Nature Ecology & Evolution 2: 1065–1070. 10.1038/s41559-018-0558-7. 29784981

[ece373458-bib-0021] Gottdenker, N. L. , D. G. Streicker , C. L. Faust , and C. R. Carroll . 2014. “Anthropogenic Land Use Change and Infectious Diseases: A Review of the Evidence.” EcoHealth 11: 619–632. 10.1007/s10393-014-0941-z. 24854248

[ece373458-bib-0022] Hartig, F. 2022. “DHARMa: Residual Diagnostics for Hierarchical (Multi‐Level / Mixed) Regression Models.” R package version 0.4.6. https://CRAN.R‐project.org/package=DHARMa.

[ece373458-bib-0023] He, J. , and T. C. G. Bosch . 2022. “ *Hydra*'s Lasting Partnership With Microbes: The Key for Escaping Senescence?” Microorganisms 10: 774. 10.3390/microorganisms10040774. 35456824 PMC9028494

[ece373458-bib-0024] Jacqueline, C. , P. A. Biro , C. Beckmann , et al. 2016. “Cancer: A Disease at the Crossroads of Trade‐Offs.” Evolutionary Applications 10: 215–225. 10.1111/eva.12444. 28250806 PMC5322410

[ece373458-bib-0025] Lenth, R. V. , B. Banfai , B. Bolker , et al. 2023. “Emmeans: Estimated Marginal Means, aka Least‐Squares Means.” R package version 1.8.9. https://cran.r‐project.org/package=emmeans.

[ece373458-bib-0026] Longdon, B. , J. D. Hadfield , J. P. Day , et al. 2015. “The Causes and Consequences of Changes in Virulence Following Pathogen Host Shifts.” PLoS Pathogens 11: e1004728. 10.1371/journal.ppat.1004728. 25774803 PMC4361674

[ece373458-bib-0027] Longdon, B. , J. D. Hadfield , C. L. Webster , D. J. Obbard , and F. M. Jiggins . 2011. “Host Phylogeny Determines Viral Persistence and Replication in Novel Hosts.” PLoS Pathogens 7: e1002260. 10.1371/journal.ppat.1002260. 21966271 PMC3178573

[ece373458-bib-0028] Maier, L. , C. Stein‐Thoeringer , R. E. Ley , et al. 2024. “Integrating Research on Bacterial Pathogens and Commensals to Fight Infections—An Ecological Perspective.” Lancet Microbe 5: 100843. 10.1016/S2666-5247(24)00049-1. 38608681

[ece373458-bib-0029] Margarita, V. , P. L. Fiori , and P. Rappelli . 2020. “Impact of Symbiosis Between *Trichomonas vaginalis* and *Mycoplasma hominis* on Vaginal Dysbiosis: A Mini Review.” Frontiers in Cellular and Infection Microbiology 10: 179. 10.3389/fcimb.2020.00179. 32457847 PMC7226223

[ece373458-bib-0030] Michalakis, Y. , and M. E. Hochberg . 1994. “Parasitic Effects on Host Life‐History Traits: A Review of Recent Studies.” Parasite 1: 291–294. 10.1051/parasite/1994014291. 9140497

[ece373458-bib-0031] Millar, R. 2011. Maximum Likelihood Estimation and Inference. With Examples in R, SAS and ADMB. Wiley. 10.1002/9780470094846.

[ece373458-bib-0032] Murillo‐Rincon, A. P. , A. Klimovich , E. Pemöller , et al. 2017. “Spontaneous Body Contractions Are Modulated by the Microbiome of *Hydra* .” Scientific Reports 7: 15937. 10.1038/s41598-017-16191-x. 29162937 PMC5698334

[ece373458-bib-0033] Posit team . 2025. RStudio: Integrated Development Environment for R. Posit Software, PBC, Boston, MA. http://www.posit.co/.

[ece373458-bib-0034] Proctor, D. M. , R. A. Drummond , M. S. Lionakis , and J. A. Segre . 2023. “One Population, Multiple Lifestyles: Commensalism and Pathogenesis in the Human Mycobiome.” Cell Host & Microbe 31: 539–553. 10.1016/j.chom.2023.02.010. 37054674 PMC10155287

[ece373458-bib-0035] R Core Team . 2022. R: A Language and Environment for Statistical Computing. R Foundation for Statistical Computing, Vienna, Austria. https://www.R‐project.org/.

[ece373458-bib-0036] Rathje, K. , B. Mortzfeld , M. P. Hoeppner , J. Taubenheim , T. C. G. Bosch , and A. Klimovich . 2020. “Dynamic Interactions Within the Host‐Associated Microbiota Cause Tumor Formation in the Basal Metazoan *Hydra* .” PLoS Pathogens 16: e1008375. 10.1371/journal.ppat.1008375. 32191776 PMC7081986

[ece373458-bib-0037] Sepp, T. , B. Ujvari , P. W. Ewald , F. Thomas , and M. Giraudeau . 2019. “Urban Environment and Cancer in Wildlife: Available Evidence and Future Research Avenues.” Proceedings of the Royal Society B: Biological Sciences 286: 20182434. 10.1098/rspb.2018.2434. PMC6367167 30963883

[ece373458-bib-0038] Shostak, S. 2017. “ *Hydra*'s Complexity: Budding and Cancer.” Trends in Developmental Biology 10: 31–39.

[ece373458-bib-0039] Stepanskyy, N. , M. Pascal , K. Asselin , et al. 2025. “Ecology of Vertical Tumor Transmission in the Freshwater Cnidarian *Hydra oligactis* .” Scientific Reports 15: 5886. 10.1038/s41598-025-88895-4. 39966423 PMC11836361

[ece373458-bib-0040] Stoy, K. S. , E. M. Díaz‐Almeyda , C. Bartelt , A. Acosta , L. T. Morran , and N. M. Gerardo . 2023. “Host‐Associated Transmission Favors Transition of a Commensal Toward Antagonism.” Evolution 77: 2512–2521. 10.1093/evolut/qpad173. 37739788

[ece373458-bib-0041] Thomas, F. , J. Guégan , Y. Michalakis , and F. Renaud . 2000. “Parasites and Host Life‐History Traits: Implications for Community Ecology and Species Co‐Existence.” International Journal for Parasitology 30: 669–674. 10.1016/s0020-7519(00)00040-0. 10779584

[ece373458-bib-0042] Tissot, S. , L. Guimard , J. Meliani , et al. 2023. “The Impact of Food Availability on Tumorigenesis Is Evolutionarily Conserved.” Scientific Reports 13: 19825. 10.1038/s41598-023-46896-1. 37963956 PMC10645767

[ece373458-bib-0043] Tissot, S. , J. Meliani , J. Boutry , et al. 2024. “ *De Novo* Evolution of Transmissible Tumours in *Hydra* .” Proceedings of the Royal Society B: Biological Sciences 291: 20241636. 10.1098/rspb.2024.1636. PMC11407858 39288800

[ece373458-bib-0044] Tissot, S. , J. Meliani , M. Chee , et al. 2024. “Cancer and One Health: Tumor‐Bearing Individuals Can Act as Super Spreaders of Symbionts in Communities.” Scientific Reports 14: 21283. 10.1038/s41598-024-72171-y. 39261506 PMC11390966

[ece373458-bib-0045] Tökölyi, J. , F. Bradács , N. Hóka , et al. 2016. “Effects of Food Availability on Asexual Reproduction and Stress Tolerance Along the Fast–Slow Life History Continuum in Freshwater *Hydra* (Cnidaria: Hydrozoa).” Hydrobiologia 766: 121–133. 10.1007/s10750-015-2449-0.

[ece373458-bib-0046] Tompkins, D. M. , A. R. White , and M. Boots . 2003. “Ecological Replacement of Native Red Squirrels by Invasive Greys Driven by Disease.” Ecology Letters 6: 189–196. 10.1046/j.1461-0248.2003.00417.x.

[ece373458-bib-0047] Verma, A. , and S. Prakash . 2022. “Anthropogenic Activities and Biodiversity Threats.” International Journal of Biological Innovations 04: 94–103. 10.46505/IJBI.2022.4110.

[ece373458-bib-0048] Vittecoq, M. , B. Roche , S. P. Daoust , et al. 2013. “Cancer: A Missing Link in Ecosystem Functioning?” Trends in Ecology & Evolution 28: 628–635. 10.1016/j.tree.2013.07.005. 23972467

[ece373458-bib-0049] Vogg, M. C. , J. Ferenc , W. C. Buzgariu , et al. 2022. “The Transcription Factor Zic4 Promotes Tentacle Formation and Prevents Epithelial Transdifferentiation in *Hydra* .” Science Advances 8: eabo0694. 10.1126/sciadv.abo0694. 36563144 PMC9788771

[ece373458-bib-0050] Wang, Z.‐D. , H.‐H. Liu , Z.‐X. Ma , et al. 2017. “ *Toxoplasma Gondii* Infection in Immunocompromised Patients: A Systematic Review and Meta‐Analysis.” Frontiers in Microbiology 8: 389. 10.3389/fmicb.2017.00389. 28337191 PMC5343064

[ece373458-bib-0051] Warren, A. , and E. A. Robson . 1998. “The Identity and Occurrence of *Kerona Pediculus* (Ciliophora: Hypotrichida), a Well‐Known Epizoite of *Hydra Vulgaris* (Cnidaria: Hydrozoa).” Zoologische Verhandelingen 323: 235–245.

[ece373458-bib-0052] Yoshida, K. , T. Fujisawa , J. S. Hwang , K. Ikeo , and T. Gojobori . 2006. “Degeneration After Sexual Differentiation in *Hydra* and Its Relevance to the Evolution of Aging.” Gene, Evolutionary Genomics 385: 64–70. 10.1016/j.gene.2006.06.031. 17011141

[ece373458-bib-0053] Zuur, A. F. , E. N. Ieno , N. Walker , A. A. Saveliev , and G. M. Smith . 2009. Mixed Effects Models and Extensions in Ecology With R, Statistics for Biology and Health. Springer. 10.1007/978-0-387-87458-6.

