# Supplementary material for: Symbiont Reintroduction Alters Tumor Progression and Life‐History Traits in the Tumor‐Bearing Freshwater Cnidarian Hydra oligactis
Source: Ecol Evol. 2026 Apr 13;16(4):e73458. doi: 10.1002/ece3.73458 (PMC13071525; doi:10.1002/ece3.73458)
Supplement: Supplementary file 1 — Appendix S1: ece373461‐sup‐0001‐AppendixS1.zip. [file ECE3-16-e73458-s001.zip › Electronic supplementary material/0. Dataset-presentation.html]

Dataset presentation


# Dataset presentation

# Main dataset presentation

### Raw variables

This section describes all raw variables for ciliate.data

- Batch : batch number
- Grandparent\_id : unique identifier for the F0 hydra
- Parent\_id : unique identifier for the F1 hydra
- GP\_state : F0 hydra tumor scale
- Lignee: tumor context, Tumoral (TTH) or ControlSP (STH), before
  modifications representent lineage, Tumoral (TL) or Control (CL)
- Infected : whether the individual was infected by ciliates (TRUE or
  FALSE)
- Birthday\_parent : date of birth of the F1 hydra
- First\_bud : date of first budding (onset of asexual
  reproduction)
- Tenta : date of appearance of supernumerary tentacles
- Observation\_date : date on which the individual was observed
- week\_day : day of the week of observation
- Class/Cilite\_number\_Parent : number of ciliates on the F1 hydra,
  grouped as A (1–9), B (10–19), C (20–29), D (30+), 0 (ciliate loss)
- Bud\_number : number of buds produced
- Age\_Semaine/Semaine : age of the individual in weeks since
  birth
- Age\_Jour : age of the individual in days since birth
- Tumeur\_start : date when the tumor first appeared
- Death : date of death of the F1 individual

### Newly created variables

This section describes all newly created variables for
ciliate.data

### 1. Ciliate infection and LHT:

- DeltaFirst\_bud : number of days between birth and first budding
  (age at firs budding)
- DeltaTUM\_day : number of days between birth and tumor appearance
  (age at tumor onset)
- DeltaFirst\_Tenta : number of days between birth and first
  appearance of supernumerary tentacles (age at supernumerary tentacles
  onset)
- TumApp : tumor appearance status (TRUE if observed, FALSE if
  not)
- TentaS : presence of supernumerary tentacles (TRUE if present,
  FALSE if absent)

### 2. Ciliate infection and Budding:

- MFC/MostFrequentClass : the most commonly observed ciliate density
  class per individual per week

# Supplementary dataset 1: preliminary cleaning protocol test

Dataset from a preliminary experiment testing whether an early
water-change protocol (1 hour after feeding) affects ciliate counts.

**Variables** :

- Batch : number indicating the day on which the pretest was
  conducted.
- id : identifier of the Hydra individual.
- Count : number of ciliates observed.
- Class : number of ciliates categorized into four levels: A (1–9),
  B (10–19), C (20–29), D (30 or more).
- Feed : a categorical variable indicating whether the count was
  done before feeding or after cleaning.

# Supplementary dataset 2 : ciliate counts on removed buds

Dataset of ciliate counts observed on individual buds prior to their
removal from wells. Includes total number of ciliates and buds per
observation date and parent.

**Variables** :

- Parent\_id : unique identifier for the F1 hydra
- Observation\_date : date on which the individual was
  observed
- Total\_Ciliate\_number\_Bud : total ciliate counts observed on buds
  prior to their removal from wells
- Bud\_number : number of buds produced
