# Supplementary material for: Symbiont Reintroduction Alters Tumor Progression and Life‐History Traits in the Tumor‐Bearing Freshwater Cnidarian Hydra oligactis
Source: Ecol Evol. 2026 Apr 13;16(4):e73458. doi: 10.1002/ece3.73458 (PMC13071525; doi:10.1002/ece3.73458)
Supplement: Supplementary file 1 — Appendix S1: ece373461‐sup‐0001‐AppendixS1.zip. [file ECE3-16-e73458-s001.zip › Electronic supplementary material/0. Packages-and-their-versions.html]

Packages and software


# Packages and software

```
library(readxl)      # For reading Excel files
library(here)        # For path management
library(dplyr)       # For data manipulation
library(tidyr)       # For fill() and other tidying functions
library(ggplot2)     # For all plotting
library(glmmTMB)     # For GLMM models
library(MuMIn)       # For AICc and model weights
library(DHARMa)      # For residual diagnostics
library(sjPlot)      # For tab_model summaries
library(ggeffects)   # For marginal effects and ggpredict()
library(ggpubr)      # For combining ggplots
library(grid)        # For margin settings in ggplot
library(emmeans)     # For estimated marginal means
```

```
sessionInfo()
```

```
## R version 4.2.2 (2022-10-31 ucrt)
## Platform: x86_64-w64-mingw32/x64 (64-bit)
## Running under: Windows 10 x64 (build 26200)
## 
## Matrix products: default
## 
## locale:
## [1] LC_COLLATE=Russian_Russia.utf8  LC_CTYPE=Russian_Russia.utf8   
## [3] LC_MONETARY=Russian_Russia.utf8 LC_NUMERIC=C                   
## [5] LC_TIME=Russian_Russia.utf8    
## 
## attached base packages:
## [1] grid      stats     graphics  grDevices utils     datasets  methods  
## [8] base     
## 
## other attached packages:
##  [1] emmeans_1.8.9   ggpubr_0.6.0    ggeffects_1.3.2 sjPlot_2.8.15  
##  [5] DHARMa_0.4.6    MuMIn_1.47.5    glmmTMB_1.1.10  ggplot2_3.5.0  
##  [9] tidyr_1.3.0     dplyr_1.1.4     here_1.0.1      readxl_1.4.3   
## 
## loaded via a namespace (and not attached):
##  [1] sass_0.4.7          jsonlite_1.8.7      splines_4.2.2      
##  [4] carData_3.0-5       modelr_0.1.11       bslib_0.6.1        
##  [7] Rdpack_2.6          stats4_4.2.2        cellranger_1.1.0   
## [10] yaml_2.3.7          bayestestR_0.13.1   numDeriv_2016.8-1.1
## [13] pillar_1.9.0        backports_1.4.1     lattice_0.22-5     
## [16] glue_1.6.2          reformulas_0.4.0    digest_0.6.33      
## [19] ggsignif_0.6.4      rbibutils_2.2.16    minqa_1.2.6        
## [22] colorspace_2.1-0    sandwich_3.0-2      htmltools_0.5.7    
## [25] Matrix_1.6-3        pkgconfig_2.0.3     broom_1.0.5        
## [28] purrr_1.0.2         xtable_1.8-4        mvtnorm_1.2-4      
## [31] scales_1.3.0        lme4_1.1-35.1       tibble_3.2.1       
## [34] mgcv_1.9-0          car_3.1-2           generics_0.1.3     
## [37] sjlabelled_1.2.0    TH.data_1.1-2       cachem_1.0.8       
## [40] withr_2.5.2         TMB_1.9.11          cli_3.6.1          
## [43] survival_3.8-3      magrittr_2.0.3      estimability_1.4.1 
## [46] evaluate_0.23       fansi_1.0.5         nlme_3.1-164       
## [49] MASS_7.3-60         rstatix_0.7.2       tools_4.2.2        
## [52] lifecycle_1.0.4     multcomp_1.4-25     munsell_0.5.0      
## [55] compiler_4.2.2      jquerylib_0.1.4     rlang_1.1.2        
## [58] nloptr_2.0.3        rstudioapi_0.15.0   rmarkdown_2.25     
## [61] boot_1.3-28.1       gtable_0.3.4        codetools_0.2-19   
## [64] abind_1.4-5         sjstats_0.18.2      sjmisc_2.8.9       
## [67] R6_2.5.1            zoo_1.8-12          knitr_1.45         
## [70] performance_0.10.8  fastmap_1.1.1       utf8_1.2.4         
## [73] rprojroot_2.0.4     insight_0.19.7      Rcpp_1.0.11        
## [76] vctrs_0.6.4         tidyselect_1.2.0    xfun_0.41          
## [79] coda_0.19-4
```
