# Supplementary material for: Symbiont Reintroduction Alters Tumor Progression and Life‐History Traits in the Tumor‐Bearing Freshwater Cnidarian Hydra oligactis
Source: Ecol Evol. 2026 Apr 13;16(4):e73458. doi: 10.1002/ece3.73458 (PMC13071525; doi:10.1002/ece3.73458)
Supplement: Supplementary file 1 — Appendix S1: ece373461‐sup‐0001‐AppendixS1.zip. [file ECE3-16-e73458-s001.zip › Electronic supplementary material/Additional files/Preliminary cleaning protocol test/Cleaning.html]

Cleaning.knit


# Lexical

- Batch : number indicating the day on which the pretest was
  conducted.
- id : identifier of the Hydra individual.
- Count: number of ciliates observed.
- Class: number of ciliates categorized into four levels: A (1–9),
  B (10–19), C (20–29), D (30 or more).
- Feed: a categorical variable indicating whether the count was
  done before feeding or after cleaning.

# Packages

```
library(readxl)
```

```
## Warning: package 'readxl' was built under R version 4.2.3
```

```
library(ggplot2)
```

```
## Warning: package 'ggplot2' was built under R version 4.2.3
```

```
library(glmmTMB)
library(MuMIn)
```

```
## Warning: package 'MuMIn' was built under R version 4.2.3
```

```
library(DHARMa)
```

```
## This is DHARMa 0.4.6. For overview type '?DHARMa'. For recent changes, type news(package = 'DHARMa')
```

```
library(tidyr)
```

```
## Warning: package 'tidyr' was built under R version 4.2.3
```

```
library(dplyr)
```

```
## Warning: package 'dplyr' was built under R version 4.2.3
```

```
## 
## Attaching package: 'dplyr'
```

```
## The following objects are masked from 'package:stats':
## 
##     filter, lag
```

```
## The following objects are masked from 'package:base':
## 
##     intersect, setdiff, setequal, union
```

# Data load and transformation

```
data = read_excel("Cleaning test.xlsx")
data$Feed = factor(data$Feed, levels = c("Before", "After"))
# Convert necessary columns to factors
data$Class = as.factor(data$Class)
data$Feed = as.factor(data$Feed)
data$id = as.factor(data$id)

#For the count data analysis, we selected the classes in which ciliates could be easily counted, for example without the risk of counting the same ciliate twice.

dataGLMM = data %>%
  filter(Class %in% c("A", "B"))
```

# GLMM

#### Step 1 : determine the optimal random effect structure (REML = TRUE)

```
F1 = glmmTMB(Count ~ Feed , REML = TRUE, family = poisson(), data=dataGLMM)

F2 = glmmTMB(Count ~ Feed  + (1|id) , REML = TRUE, family = poisson(), data=dataGLMM)

F3 = glmmTMB(Count ~ Feed  + (1|Batch) , REML = TRUE, family = poisson(), data=dataGLMM)

F4 = glmmTMB(Count ~ Feed  + (1|id) + (1|Batch)  , REML = TRUE, family = poisson(), data=dataGLMM)

# Compare the performance of the models
ss = AICc(F1, F2, F3, F4) #Akaike Information Criterion (AIC) 
delta_ss = ss$AIC - min(ss$AIC) #Delta AIC = difference between the AIC of the best model and all other model
weight_ss = Weights(ss) #AIC Weight = The probability that a model is the best out of the set of fitted model

# Create a summary table
ss$delta_ss  = delta_ss 
ss$weight_ss = round(weight_ss, digit = 2)
ss #F2 = 0.75
```

```
##    df     AICc delta_ss weight_ss
## F1  2 462.8170 137.0497      0.00
## F2  3 325.7674   0.0000      0.75
## F3  3 464.7862 139.0189      0.00
## F4  4 328.0124   2.2450      0.25
```

#### Step 2 : determine the optimal fixed effect structure (REML = FALSE)

```
F2_1 = glmmTMB(Count ~ Feed  + (1|id), REML = FALSE, family = poisson(), data=dataGLMM)

F2_2 = glmmTMB(Count ~ 1 + (1|id), REML = FALSE, family = poisson(), data=dataGLMM)

# Compare the performance of the models
ss = AICc(F2_1, F2_2) #Akaike Information Criterion (AIC) 
delta_ss = ss$AIC - min(ss$AIC) #Delta AIC = difference between the AIC of the best model and all other model
weight_ss = Weights(ss) #AIC Weight = The probability that a model is the best out of the set of fitted model

# Create a summary table
ss$delta_ss  = delta_ss 
ss$weight_ss = round(weight_ss, digit = 2)
ss #F2_2 = 0.75
```

```
##      df     AICc delta_ss weight_ss
## F2_1  3 320.8417 2.155594      0.25
## F2_2  2 318.6861 0.000000      0.75
```

#### Step 3 : refit the final model using REML estimator (REML = TRUE)

```
F2_2 = glmmTMB(Count ~ 1 + (1|id), REML = TRUE, family = poisson(), data=dataGLMM)
```

#### Compute the residuals of the model

```
resids = simulateResiduals(F2_2, plot=TRUE)
```

#### Results of the model

```
summary(F2_2)
```

```
##  Family: poisson  ( log )
## Formula:          Count ~ 1 + (1 | id)
## Data: dataGLMM
## 
##      AIC      BIC   logLik deviance df.resid 
##    320.7    325.1   -158.3    316.7       64 
## 
## Random effects:
## 
## Conditional model:
##  Groups Name        Variance Std.Dev.
##  id     (Intercept) 0.5517   0.7428  
## Number of obs: 66, groups:  id, 33
## 
## Conditional model:
##             Estimate Std. Error z value Pr(>|z|)    
## (Intercept)    1.557      0.144   10.81   <2e-16 ***
## ---
## Signif. codes:  0 '***' 0.001 '**' 0.01 '*' 0.05 '.' 0.1 ' ' 1
```

# Class check

We will also check if classes change globally

```
summary_data = data %>%
  dplyr::group_by(Class, Feed) %>%
  dplyr::summarize(Frequency = dplyr::n(), .groups = "drop")

# Create the bar plot
ggplot(summary_data, aes(x = Class, y = Frequency, fill = Feed)) +
  geom_bar(stat = "identity", position = "dodge") +
  scale_fill_manual(values = c("Before" = "skyblue", "After" = "lightgreen")) +
  labs(title = "Class Distribution before and after Feeding",
       x = "Class",
       y = "Frequency",
       fill = "Feeding") +
  theme_minimal()
```
