# Supplementary material for: Symbiont Reintroduction Alters Tumor Progression and Life‐History Traits in the Tumor‐Bearing Freshwater Cnidarian Hydra oligactis
Source: Ecol Evol. 2026 Apr 13;16(4):e73458. doi: 10.1002/ece3.73458 (PMC13071525; doi:10.1002/ece3.73458)
Supplement: Supplementary file 1 — Appendix S1: ece373461‐sup‐0001‐AppendixS1.zip. [file ECE3-16-e73458-s001.zip › Electronic supplementary material/README.html]

README file


# README file

# Associated article

**Article title** : Symbiont reintroduction alters tumor
progression and life-history traits in tumor-bearing freshwater
cnidarian *Hydra oligactis*

# Associated data

All supplementary files are contained within the **Electronic
supplementary material** folder. The folder structure described
below uses paths relative to this main directory.

### Raw data file

The raw data file *ciliate\_data.xlsx* is available in the
**Code and data** folder, within the **Raw
data** subfolder.

### Variables description

All variables are described in ***Dataset
presentation.html***, as well as in the
**Lexical** section at the beginning of each script.

### Packages and software

All packages and software used, along with their versions, are
provided in ***Packages and their
versions.html***.

### R Scripts

All R scripts are available in the **Code and data**
folder, within the **R Scripts** subfolder. The folder
contains two R Markdown (.Rmd) files: **Ciliate infection
and\_LHT.rmd**, which contains the analysis of all traits except
budding rate, and **Ciliate infection and Budding.rmd,**
which contains the analysis of budding rate.

### Figures

The **Figures** folder contains high-resolution image
files corresponding to the figures in the main article, and the
**Figures rds** folder in the **Code and
data** directory contains the `.rds` files used to
create Figure 4 in the article.

### Additional files

The **Additional files** folder contains the following
subfolders:

- **Class illustration** - video illustrations of
  different ciliate classes.
- **Ciliate counts on removed buds** - dataset of
  ciliate counts observed on individual buds prior to their
  removal.
- **Preliminary cleaning protocol test** - dataset and
  analysis from the preliminary experiment testing whether an early
  water-change protocol (1 hour after feeding) affects ciliate
  counts.
