# Supplementary figures and images for: Symbiont Reintroduction Alters Tumor Progression and Life‐History Traits in the Tumor‐Bearing Freshwater Cnidarian Hydra oligactis
Source: Ecol Evol. 2026 Apr 13;16(4):e73458. doi: 10.1002/ece3.73458 (PMC13071525; doi:10.1002/ece3.73458)

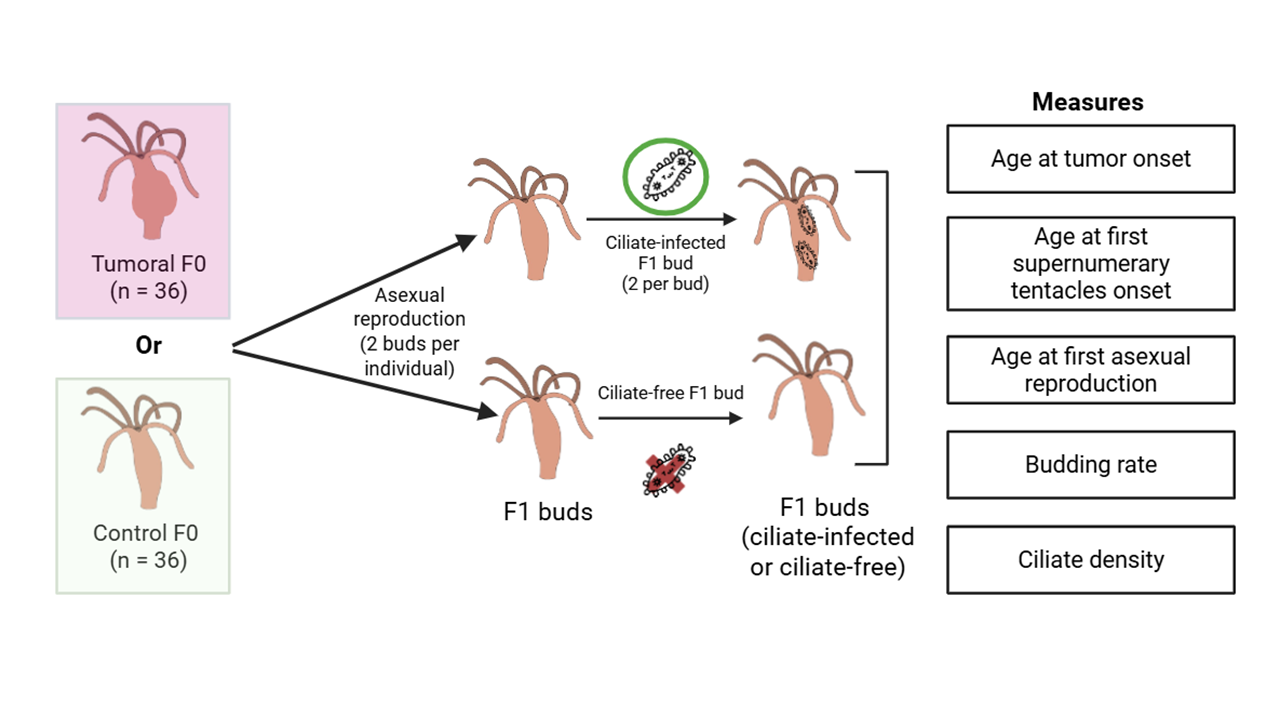

Supplement: Supplementary file 1 — Appendix S1: ece373461‐sup‐0001‐AppendixS1.zip. [file ECE3-16-e73458-s001.zip › Electronic supplementary material/Figures/Fig1.PNG]

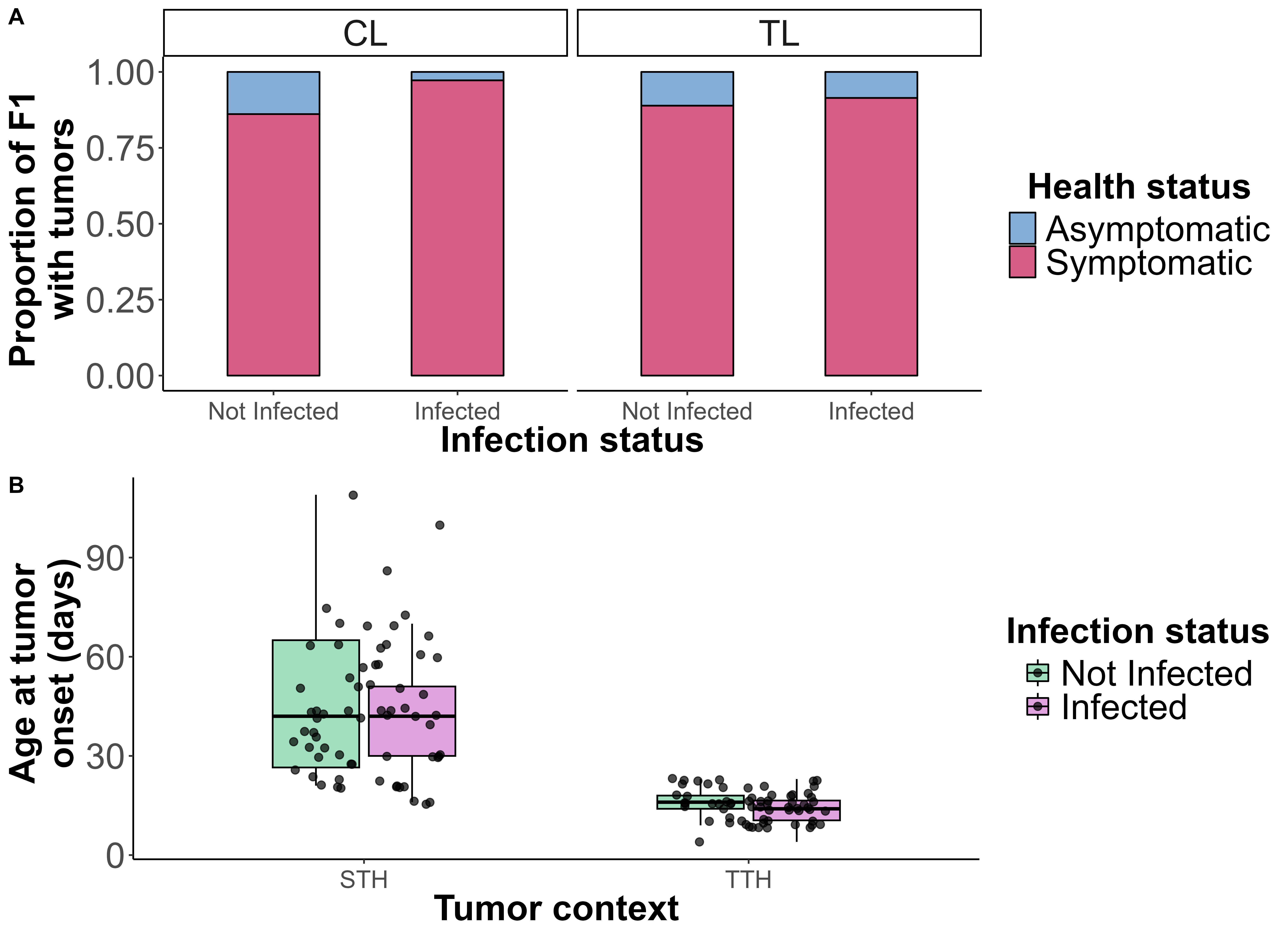

Supplement: Supplementary file 1 — Appendix S1: ece373461‐sup‐0001‐AppendixS1.zip. [file ECE3-16-e73458-s001.zip › Electronic supplementary material/Figures/Fig2.png]

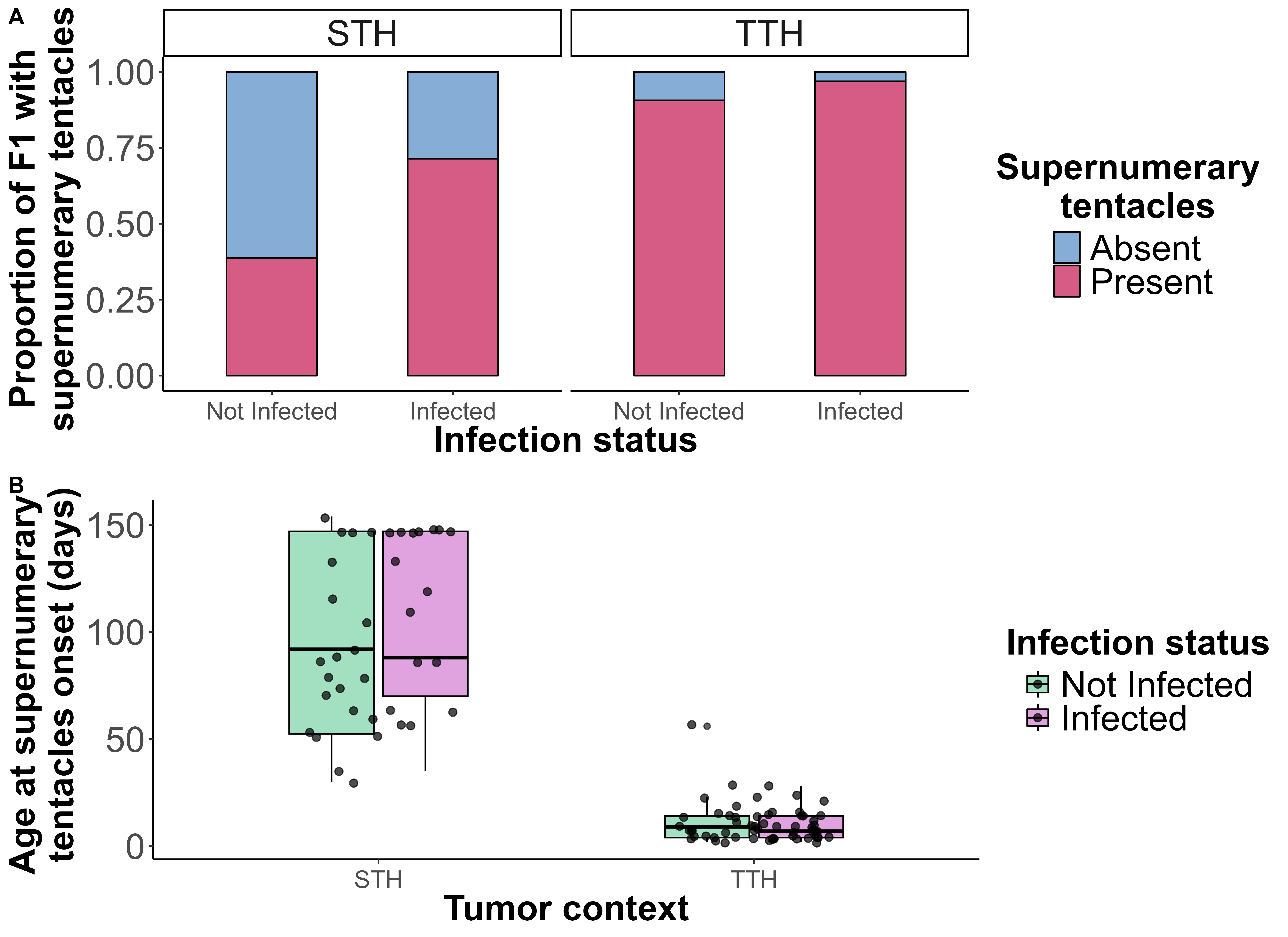

Supplement: Supplementary file 1 — Appendix S1: ece373461‐sup‐0001‐AppendixS1.zip. [file ECE3-16-e73458-s001.zip › Electronic supplementary material/Figures/Fig3.png]

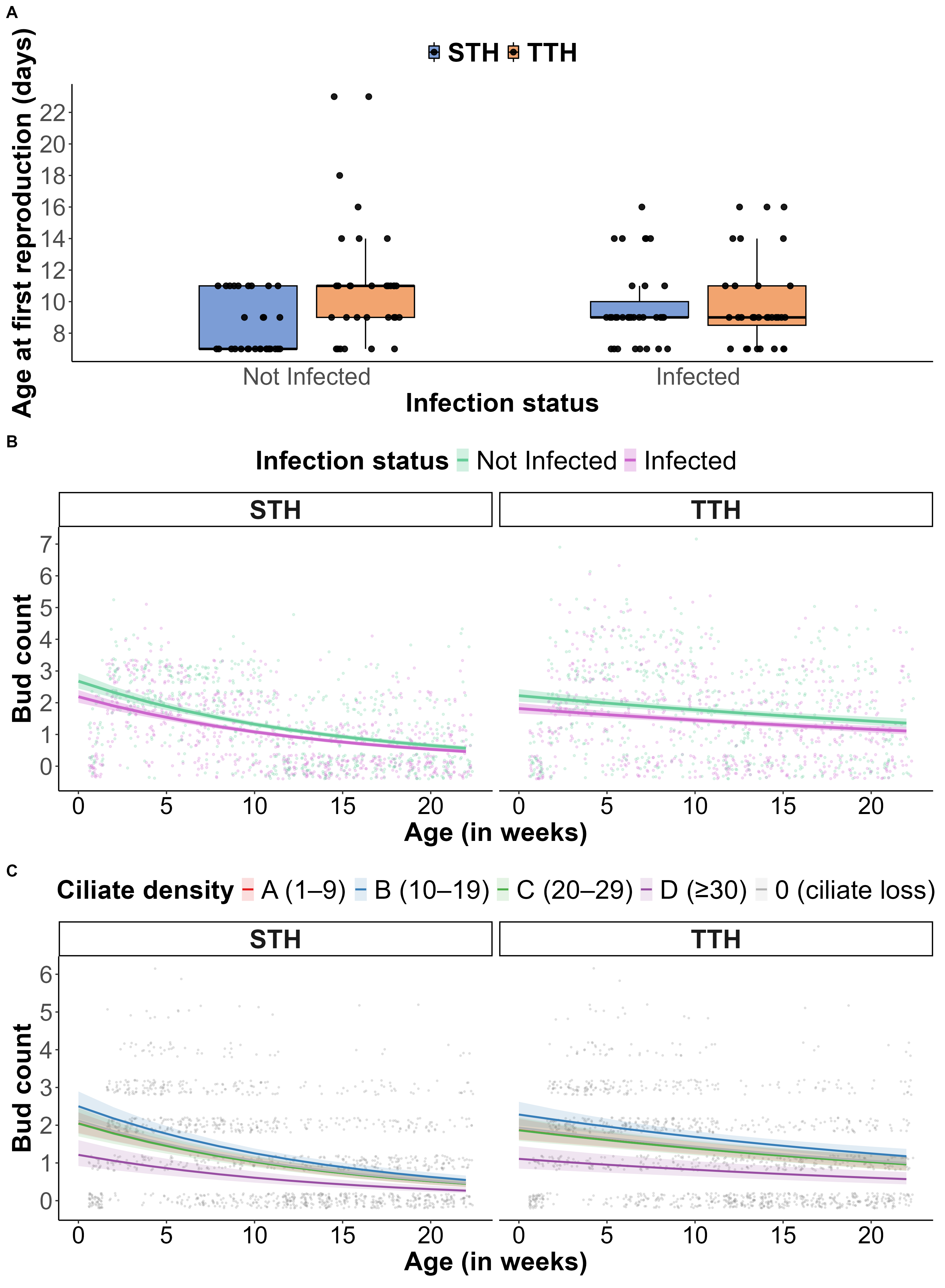

Supplement: Supplementary file 1 — Appendix S1: ece373461‐sup‐0001‐AppendixS1.zip. [file ECE3-16-e73458-s001.zip › Electronic supplementary material/Figures/Fig4.png]
